# Isolating Influenza RNA from Clinical Samples Using Microfluidic Oil-Water Interfaces

**DOI:** 10.1371/journal.pone.0149522

**Published:** 2016-02-17

**Authors:** Francis R. Cui, Jingjing Wang, Steven M. Opal, Anubhav Tripathi

**Affiliations:** 1 Center for Biomedical Engineering, School of Engineering, Brown University, Providence, Rhode Island, United States of America; 2 Warren Alpert Medical School, Brown University, Providence, Rhode Island, United States of America; 3 Memorial Hospital of Rhode Island, Pawtucket, Rhode Island, United States of America; Naval Research Laboratory, UNITED STATES

## Abstract

The effective and robust separation of biomolecules of interest from patient samples is an essential step in diagnostic applications. We present a platform for the fast extraction of nucleic acids from clinical specimens utilizing paramagnetic PMPs, an oil-water interface, a small permanent magnet and a microfluidic channel to separate and purify captured nucleic acids from lysate in less than one minute, circumventing the need for multiple washing steps and greatly simplifying and expediting the purification procedure. Our device was able to isolate influenza RNA from clinical nasopharyngeal swab samples with high efficiency when compared to the Ambion® MagMAX^TM^ Viral RNA Isolation Kit, sufficiently separating nucleic acid analytes from PCR-inhibiting contaminants within the lysate while also critically maintaining high integrity of the viral genome. We find that this design has great potential for rapid, efficient and sensitive nucleic acid separation from patient sample.

## Introduction

The extraction of DNA, RNA and protein from biological specimens is the first and most essential step for numerous biomedical applications[1] including the diagnosis of viral and bacterial infections[2], protein expression analysis, the characterization of tumor markers[3] and other basic researches[4]. The high quality of purified nucleic acids (NA) from patient samples is critical for their efficient downstream processing and sensitive analysis, especially for point-of-care (POC) molecular diagnostics[5]. Traditional methods for nucleic acid isolation can be time-consuming and labor-intensive, often requiring multiple washing steps, centrifugation and reagents that are not readily found in most clinical settings where rapid and accurate diagnostics are greatly needed. The development of a micro total analysis system (μTAS) is therefore limited by the challenge of streamlined sample preparation and the adaptation of traditional macroscale techniques to the micro-scale[6].

Many commercial kits (Qiagen, Ambion, Promega and others) have been developed for the solid phase extraction (SPE) of nucleic acids by utilizing paramagnetic particles (PMPs) or columns that selectively bind DNA and/or RNA[7–9]. Though they offer the convenience of ready-made reagents, most of these protocols still require repeated centrifugation or washing steps. In the demand for high throughput sample processing, automated systems have been designed to replace labor-intensive manual procedures[9]. Though these systems are accurate and reliable, they remain expensive and not scalable, and therefore inapplicable for low-resource settings lacking the requisite laboratory equipment and necessary expertise to perform these assays. A simple, rapid, and sensitive extraction technique is needed for the sample-in, result-out platform in POC settings.

Recent years have thus seen the emergence of microfluidic isolation platforms utilizing SPE, which can be implemented more easily than its liquid-liquid counterpart. These technologies typically involve the flow of lysate through micropillars[10], silica bead/sol-gel suspensions[11] or polymeric monoliths[12], followed by the subsequent washing and elution of adsorbed NA. Though these offer an effective means of isolation, they require external pumps and other hardware, making them ill-suited for POC diagnostic use. More promising and practical is the burgeoning technological subset of stationary microfluidics[13], which relies on the movement of PMPs rather than pressure-driven flow[14]. By incorporating hydrophobic phases within a microfluidic system, adsorbed nucleic acids can be separated from lysate using just a permanent magnet and the high interfacial tension between the two phases. An immiscible phase in the form of air[15, 16] or oil[17] can therefore serve as a valve or filter by pinching off the PMPs from the rest of the lysate.

With a simple design, facile operation and no need for peripheral equipment, immiscible phase filtration has potential to spawn technologies for low resource, POC use as well as for high throughput sample preparation. In the area of NA extraction, Bordelon et al. have constructed a promising design using air surface tension valves to isolate respiratory syncytial virus RNA[15]. Recovery rates, however, were reported to be low compared to the commercial extraction kit, and the device length (at 61 cm long) made it impractical. Den Dulk et al. have produced a similar design by patterning hydrophilic and hydrophobic surfaces onto a glass chip[16]. Oil and liquid wax have also been implemented in technologies such as the IFAST (Immiscible Filtration Assisted by Surface Tension) devices to serve as a valve between lysate and elution chambers[18–21]. Though these studies have been able to achieve sensitive DNA/RNA isolation, results drawn from clinical samples have heretofore been lacking.

In this report we describe a stationary microfluidic method for extracting and recovering RNA from patient samples using oil-water interfaces. Our oil chip device differentiates itself by being a closed microfluidic system, which in the POC setting is a necessity to prevent contamination. This device was then tested by extracting influenza RNA from nasopharyngeal swab specimens, representing the first instance of a technique of this kind being used on clinical samples. After mixing the samples with silica PMPs and lysis/binding solution, the PMPs were pulled through an oil-filled microchannel and into a well containing elution buffer. Sample extractions were performed in tandem with the commercial Ambion kit, with the aim of achieving equivalent sensitivity. Eluted total RNA was then quantified with a UV-vis spectrometer, vRNA integrity was confirmed by RNA gel electrophoresis and extraction efficiency was assessed by RT-qPCR.

## Materials and Methods

### Microfluidic chip design

**Fig 1:**
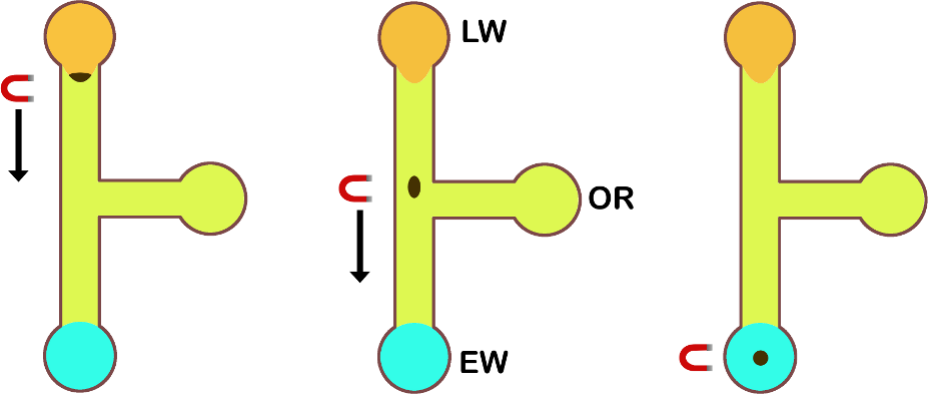
Channel design and operation. Left: the PMP/lysate suspension is pipetted into the lysate well (LW) and a permanent magnet draws the PMPs to coalesce at the oil-water interface. Middle: moving the magnet slowly towards the elution well (EW) causes the PMPs to penetrate the oil phase. Right: the PMP aggregate is then pulled into the EW, the contents of which are subsequently removed and vortexed to elute the RNA.

The device consists of three wells joined by two channels intersecting to form a T-junction (Fig 1). The longer channel connects the lysate well (LW) and the elution well (EW), while the shorter channel bisects the longer channel and joins the oil reservoir (OR). The channels are 1.25 mm wide and 150 μm deep, allowing for the transfer of a greater amount of PMPs. The relatively large cross-sectional area of the channel is critical as traversal across the immiscible oil-water barrier requires a sufficient magnetic dipole force, thus necessitating the PMPs to cross the interface as a single aggregate. Various channel lengths were tested and optimized for PMP transfer efficiency, defined as the ratio of PMPs eluted to PMPs added.

### Device fabrication and preparation

The device is composed of a microscope slide bonded to solid polydimethysiloxane (PDMS) chip, fabricated via soft lithography techniques. PDMS precursor and curing agent (Dow Corning, Midland, MI) were mixed in a 10:1 mass ratio, degassed for 1 hr, poured over a solid SU-8 structure and allowed to cure for 1 hr at 70°C. The solidified polymer was then excised, hole-punched and finally bonded to a microscope slide via oxygen plasma treatment with a high frequency generator (Electro-Technic Products, Chicago, IL). The chip was then incubated at 70°C for an additional 30 min to enhance bonding.

Upon complete bonding, the chip was rinsed with RNAse decontamination solution (Integrated DNA Technologies, Coralville, IA), followed by several subsequent washes with nuclease-free water. The channels were then cleared of liquid with high-pressure nitrogen gas. Once decontaminated, the channels were filled with olive oil by pipetting approximately 10 μL into the OR and allowing the oil to spread under capillary action. The particular olive oil used in this device had a viscosity of 93 cP at 22°C, as measured by a rheometer (Texas Instruments, New Castle, DE); as well as an interfacial tension of 6.45 mN/m with Ambion lysis-binding solution, as measured by a goniometer (Kruss, Denmark). 10 μL of priming solution was then pipetted into each aqueous-phase well (LW and EW). The priming solution consisted of 1 mg/mL bovine serum albumin (New England Biolabs, Ipswich, MA) and 0.01% Tween 20 (Sigma Aldrich, St. Louis, MO) in nuclease-free water, with the intention of blocking non-specific binding sites on the channel surfaces, as well as decreasing PMP adsorption to the glass bottom. Once primed, the chip was incubated overnight at 4°C.

### Operation principle and procedure

For RNA separation, we adopted the procedure for the Ambion® MagMAX^TM^ Viral RNA Isolation Kit (Thermo Fischer Scientific, Waltham, MA), eschewing the wash steps and scaling down reagent volumes. The protocol begins with single-step cell lysis and RNA-PMP binding, which involves the mixture of a lysis/binding solution (containing GuSCN, isopropyl alcohol and carrier RNA to facilitate nucleic acid precipitation), biological sample and PMP mix. After 4 min of gentle mixing, the PMPs with adsorbed RNA are drawn to the bottom of the tube with a small, cylindrical (0.5-in diameter, 0.375-in thick), grade N52 neodymium magnet (K&J Magnetics, Jamison, PA) and pipetted out along with 10 μL of lysate, which is then added to the LW after 10 μL of priming solution has been aspirated. This step serves to preconcentrate the PMPs before loading, though a modified device with a larger LW could be used to the same effect. Similarly, the priming solution in the EW is replaced with an equal volume of elution buffer (an aqueous, low salt solution).

Once in the LW, the PMPs are coerced by the magnet to form a compact aggregate, which can then penetrate the oil-water interface and enter the oil-filled channel. Upon interface traversal, the PMPs will remain in a tight clump and glide easily along the channel, following the movement of the magnet. Optimal PMP transfer typically occurs when the speed of the magnet below 1 mm/s; greater speeds may lead to break-up of the PMP aggregate. This process of PMP aggregation, interface penetration and immiscible phase traversal can be completed in 0.5–2 minutes. Once pulled into the EW, the PMPs along with 10 μL of elution buffer are drawn out and vortexed vigorously to elute the RNA from the silica surfaces. Table 1 details the time and reagent volume saved with this procedure compared to a commercial kit protocol.

**Table 1:** Summary of protocols. Comparison of MagMAX and oil chip reagents and time required to complete both procedures. First and second washes are both repeated for a total of four washes.

	Sample (μL)	Lyse/bind soln. (μL)	1^st^ wash (μL)	2^nd^ wash (μL)	Elution buffer (μL)	Time elapsed (min)
MagMAX	100	200	75	112	25	31
Oil chip	40	80	0	0	10	11

### Clinical influenza RNA extraction

Serial clinical samples were obtained from patients presenting with influenza-like symptoms to Memorial Hospital of RI during the 2013–2014 winter influenza season. These nasopharyngeal swab samples, which were suspended in viral transport medium and heat-inactivated, were assigned a randomized code number and then patient de-identified as per Institutional Review Board protocol, which allowed for maintenance of patient confidentiality and for a waiver of informed consent. RNA extraction was performed using both the commercially available MagMAX kit and our oil chip. Eluted samples were then analyzed with a Nanodrop spectrophotometer (Thermo Fischer Scientific, Waltham, MA) for analyte concentration and purity. Viral RNA integrity was assessed with the Bioanalyzer RNA 6000 Pico assay (Agilent, Santa Clara, CA).

RT-qPCR was performed on a PikoReal 24 Real-Time PCR System (Thermo Fischer Scientific, Waltham, MA) following the CDC protocol for swine influenza A (H1N1) using the SuperScript III One-Step RT-PCR kit (Thermo Fischer Scientific, Waltham, MA) with all-type influenza A forward (5’-GACCRATCCTGTCACCTCTGAC-3’) and reverse (5’-AGGGCATTYTGGACAAAKCGTCTA-3’) primers and TaqMan probe (5’-TGCAGTCCTCGCTCACTGGGCACG-3’) (Integrated DNA Technologies, Coralville, IA)[22]. Purified H1N1 RNA from strain A/Virginia/ATCC2/2009 (ATCC, Manassas, VA) was used as a positive template control and amplified to make serial dilutions for the standard curve.

## Results

**Fig 2:**
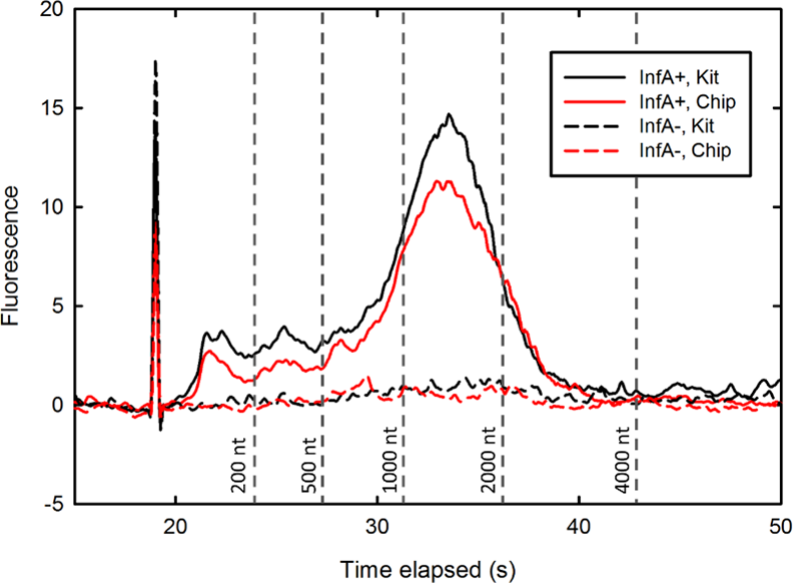
Size distribution of extracted RNA. Electropherogram of Bioanalyzer RNA 6000 Pico gel assaying processed samples 361 (influenza-positive) and 10 (influenza-negative). vRNA extracted by both the MagMAX kit and oil chip exhibits a smear from 900 to 2500 nt, characteristic of the segmented RNA influenza genome.

Viral RNA extracted from clinical nasal swab samples was examined via gel electrophoresis to initially confirm the successful isolation of influenza RNA. Samples isolated by both kit and chip methodologies were run on an RNA 6000 Pico gel chip (Fig 2) and produced similar profiles, with one smear ranging from 900 to 2500 nt, which concurs with the size range of the influenza genome[23]. A sample from a healthy patient (influenza A/B negative) was processed in the same manner and run as a negative control. Kit and chip RNA produced bands of comparable width and signal intensity, suggesting that the oil chip method is capable of extracting nearly equal the amount of RNA without additional degradation.

Device efficacy was assessed by performing vRNA extraction on clinical nasopharyngeal swab samples and measuring total RNA yield using spectrophotometry. As these samples are relatively cell-free, the solid phase extraction protocol for vRNA requires the addition of carrier RNA to aid in the precipitation of nucleic acid out of solution and onto the PMPs. The absorbance at 260 nm is therefore almost entirely due to captured carrier RNA, which significantly outweighs any vRNA in the sample. Nevertheless, these measurements are good indicators of device sensitivity; high OD260 values may suggest less bead loss and greater RNA precipitation, while lower values may indicate higher bead loss or reduced precipitation.With the commercial kit and oil chip protocols we were able to extract a mean total RNA concentration of 15.6 ± 4.4 ng/μL and 12.7 ± 4.6 ng/μL, respectively (uncertainties represent standard deviations) over 28 distinct patient samples. Defining the spectrophotometrically determined percent efficiency E_tot_ as the ratio of OD260 measurements yielded by the oil chip and commercial kit (i.e., E_tot_ = 100*[chip OD260]/[kit OD 260] per individual patient sample), the oil chip on average performs at E_tot_ = 89.1% (S1 Table). This simple device can thus extract a nearly equivalent amount of total RNA per sample volume input in roughly one third of the time.

Solid phase extraction contains alcohol and chaotropic reagents such as GuSCN that may inhibit PCR. This issue is typically addressed by the repeated washing of the silica surfaces with a nonpolar solvent. While passing the PMPs through a hydrophobic barrier filters out most contaminants, a small volume of lysate is inevitably carried over as the PMPs remain wetted by the aqueous phase during their passage through the oil. To assess the functionality of isolated sample, as well as to quantify vRNA capture efficiency, we performed RT-qPCR following the CDC 2009 H1N1 protocol, with universal influenza A primers that targeted a 106 bp amplicon located on the *M1/M2* gene segment. Standard and sample curves for patient samples 177, 78 and 193 (S1 Fig) reveal an amplification efficiency of 99.8% percent and high end-point sample amplification. A DNA electropherogram of amplification products (S2 Fig) shows no signal due to contamination and erroneous amplification.

**Fig 3:**
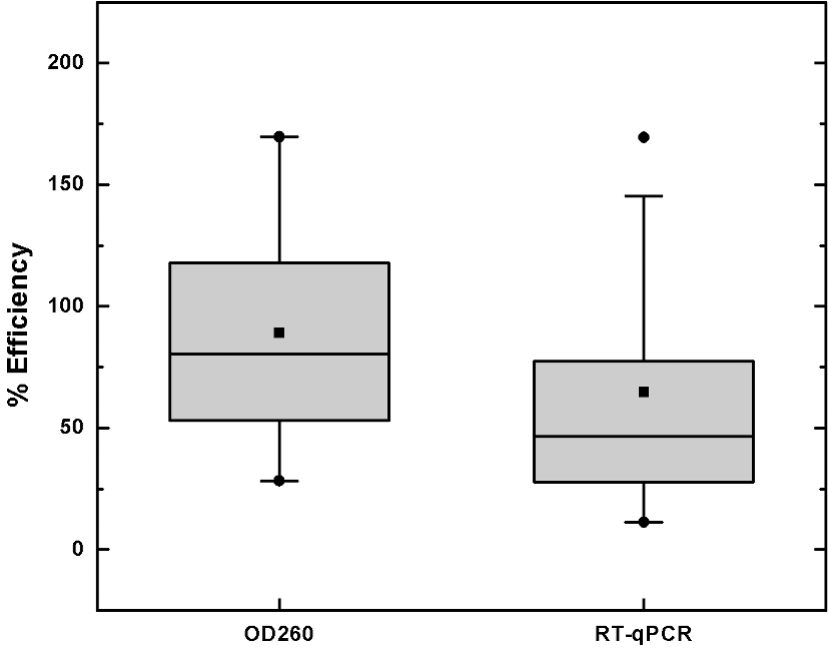
Summary of patient sample RNA extraction. Box plots show the distribution of oil chip efficiency over all 28 patient samples. Squares represent averages, circles represent the minimum and maximum values.

The ratio of chip extracted-to-kit extraction vRNA yield, or E_vir_ for each patient sample was calculated as E_vir_ = 2^Δ*Ct*^, where ΔCt is the difference in threshold cycle between kit-extracted and chip-extracted RNA samples (S2 Table). Viral loads given by each methodology were also quantified. The results of both UV-vis and RT-qPCR measurements are summarized in Fig 3. Real-time amplification indicates an average vRNA extraction efficiency E_vir_ of 64.9% and a median efficiency of 47.5%. This lower value (compared with total RNA extraction efficiency measured by absorbance) is likely due to the small amount of inhibitors that are carried over with the PMPs during extraction. E_vir_ was also measured for samples spiked with known amounts of input template. Amplicon was spiked into phosphate buffered saline at various concentrations (ranging from 10^8^ to 10^6^ copies/mL) and processed via kit and oil chip, giving average template recoveries of 36.2 ± 4.4% and 21.9 ± 2.4%, respectively, to give E_vir_ = 60.5 ± 9.8%.

## Discussion

Overall, the successful operation of our oil chip device came about without the need for exhaustive optimization, though a comparison of the average E_tot_ and E_vir_ values suggests that there is some inhibition of PCR occurring in the chip-extracted samples. One simple method for determining RNA purity is to quantify the 260/230 optical density ratio, which can signal the presence of lysing agents present in the eluted RNA. Chip-extracted RNA typically gave 260/230 ratios between 0.03 and 0.07, indicating that some amount of lysate was carried over with the PMPs. This may explain why the qPCR-determined viral RNA yield is lower than the OD-determined total RNA yield. It is likely that the oil chip is able to extract vRNA with the same efficiency as with total RNA, but the presence of isopropanol and GuSCN in the eluted sample may inhibit amplification and lead to lower E_vir_. Nevertheless, if used within a point of care diagnostic device that is mainly concerned with obtaining a positive/negative result, the oil chip technique will still extract sufficient RNA in roughly one third of the time, compared to the commercial kit.

The large variances of E_tot_ and E_vir_ may be explained by the nature of the clinical samples. Different amounts of inflammatory cells and varying viscosities between the patient samples may have differentially affected the operation of the oil chip. Large E_vir_ values, however, typically corresponded with large E_tot_ values, as seen in four patient samples with among the highest E_vir_ values (Sample IDs: 78, 179, 327, 439). Additionally, reduction in the extent of RNA shearing may have contributed to variation. Occasionally but not consistently, peak shifting in RNA electropherograms were observed for chip-extracted samples, such that the 900–2500 nt band became skewed towards the right (i.e., longer segments). Because the oil chip procedure requires much less vigorous vortexing, a possible advantageous outcome might be gentler treatment of the RNA, which is prone to shearing.

We found the oil chip to be flexible in its design and operation parameters; different magnet strengths, oil types (in addition to olive oil, vegetable and canola oil were also tested) and channel dimensions were implemented to roughly the same effect. Ultimately, device efficacy depends on the movement of PMPs as one aggregate across the oil-water interface. Once this step is accomplished, the captured analyte can be separated with (as in the case of influenza RNA from patient sample) negligible contaminant carryover. PMP mobility and aggregation is thus critical to successful separation. For a given channel geometry there exists an optimum PMP aggregate size that ensures successful penetration; too few PMPs and there is an insufficient magnetic dipole force to overcome interfacial capillary forces; too many, and the PMP aggregate will fill the width of the channel and simply pull the interface with it as the magnet is moved, preventing the oil phase from pinching off the aqueous tail. The magnet should therefore be held for a certain amount of time (roughly 30 seconds for this device) in order to coalesce the PMPs. The time scale for this event depends on the priming solution used, magnet strength and magnetic susceptibility of the PMPs.

As the PMP aggregate moves through the oil it travels as a closed aqueous microenvironment. Because it is surrounded by an immiscible phase, it is energetically unfavorable for the aggregate to partition or break up, thus facilitating the transport of PMPs through the oil. This scheme could potentially be used for other separation applications that involve the capture of analyte by PMPs (e.g., antibody- or aptamer-functionalized PMPs), as the local chemical environment of the PMPs remains constant even as they enter the oil, thus preventing unwanted or premature elution. Furthermore, more stringent purification or step-wise processing with various labels (such as in an ELISA) may be achieved by multiple oil-water barriers in series. Insufficient interfacial tension between the lysate and oil can however lead to excessive stretching of the oil-water interface and the formation of an aqueous phase bridge connecting the LW and EW. It is therefore necessary to fine-tune the surfactant concentration to ensure a balance between PMP mobility and high surface tension, thus dictating the balance between PMP transfer efficiency and carry-over minimization. As discussed by Berry et al. [18], the interfacial tensions of the lysate-oil (γ_LO_), lysate-substrate (γ_LS_) and oil-substrate (γ_OS_) interfaces determine whether complete separation of the PMPs and lysate occurs. To prevent the formation of an aqueous bridge, high γ_LO_ and γ_LS_ must be utilized to favor the pinching off of the PMP aggregate.

## Conclusions

We have demonstrated with a simple design the efficient separation of RNA from patient sample without the need for washing and centrifugation. Our device, when compared to the Ambion MagMAX kit, can be run in a third of the time with nearly equivalent efficacy, using only a pipette and permanent magnet. Furthermore it requires less volume of reagents and sample, and can be applied to not just solid phase extraction but likely any separation technique involving analyte capture on PMPs. With the oil chip, we were able to isolate influenza RNA from clinical samples at high concentration while also maintaining vRNA integrity. The carryover of lysate had a small inhibitory effect on amplification, as shown by the slight drop in vRNA extraction efficiency compared to total RNA extraction efficiency; however, the overall performance was promising given the rapidity and ease of operating the oil chip. We conclude that this technique has great potential for both point-of-care as well as high-throughput applications.

## Supporting Information

S1 Fig RT-PCR standard curve. a) The standard curve was formed from serial dilutions ranging from 10^9^ to 10^5^ copies/mL. Amplification efficiency was calculated to be 99.8%. b) RT-qPCR amplification plot for samples 177, 78 and 193.
(TIF)

S2 Fig RT-PCR of patient samples. Electropherogram of Bioanalyzer DNA 1000 gel assaying RT-PCR products of processed patient samples 590, 177 and 474, the last of which tested negative for influenza A. cDNA concentrations (ng/μL) are given next to respective peaks.
(TIF)

S3 Fig Peak shift in extracted RNA. RNA Electropherogram for patient sample 193 processed both by kit and oil chip method. The peak shift towards the right for the oil chip sample was observed.
(TIF)

S1 Table Total RNA yield from both methods for 28 separate clinical nasal swab samples, given by optical density measurements. E_tot_ for Column 4 is calculated as the ratio of OD260 measurements of chip-extracted RNA to kit-extracted RNA.
(DOC)

S2 Table Quantitative RT-PCR was performed on kit- and chip-extracted samples. (DOC)
