# Supplementary figures and images for: Isolating Influenza RNA from Clinical Samples Using Microfluidic Oil-Water Interfaces
Source: PLoS One. 2016 Feb 17;11(2):e0149522. doi: 10.1371/journal.pone.0149522 (PMC4757531; doi:10.1371/journal.pone.0149522)

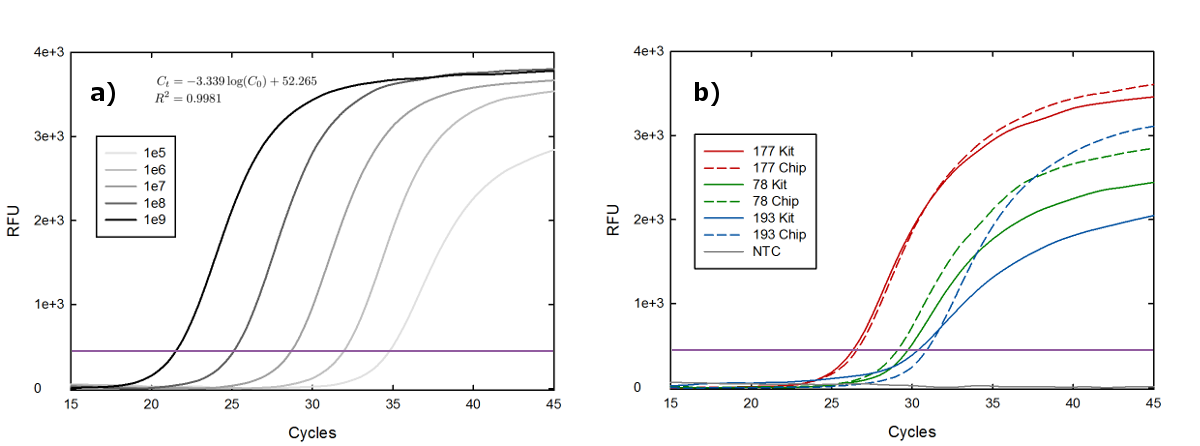

Supplement: S1 Fig — a) The standard curve was formed from serial dilutions ranging from 109 to 105 copies/mL. Amplification efficiency was calculated to be 99.8%. b) RT-qPCR amplification plot for samples 177, 78 and 193. (TIF) [file pone.0149522.s001.TIF]
