# Supplementary material for: Isolating Influenza RNA from Clinical Samples Using Microfluidic Oil-Water Interfaces
Source: PLoS One. 2016 Feb 17;11(2):e0149522. doi: 10.1371/journal.pone.0149522 (PMC4757531; doi:10.1371/journal.pone.0149522)
Supplement: S1 Table — Etot for Column 4 is calculated as the ratio of OD260 measurements of chip-extracted RNA to kit-extracted RNA. (DOC) [file pone.0149522.s004.doc]

Table S1

| Sample ID | **Kit (ng/μL)** | **Oil chip (ng/μL)** | **E_tot_ (%)** |
| --- | --- | --- | --- |
| **78** | 18.0 | 23.2 | 128.8 |
| **86** | 17.1 | 14.1 | 82.2 |
| **98** | 10.1 | 11.9 | 117.8 |
| **103** | 18.9 | 9.8 | 52.1 |
| **134** | 8.9 | 15.1 | 169.7 |
| **177** | 20.3 | 16.7 | 82.2 |
| **179** | 13.6 | 18.9 | 139.0 |
| **193** | 14.7 | 24.5 | 167.5 |
| **204** | 14.2 | 7.5 | 53.2 |
| **218** | 13.5 | 10.8 | 80.5 |
| **232** | 12.2 | 11.9 | 97.5 |
| **254** | 17.9 | 5.3 | 29.6 |
| **286** | 18.5 | 5.2 | 28.3 |
| **318** | 12.9 | 14.2 | 110.1 |
| **327** | 6.6 | 8.1 | 123.5 |
| **349** | 22.0 | 10.0 | 45.5 |
| **401** | 20.9 | 15.7 | 75.4 |
| **412** | 11.4 | 16.9 | 148.2 |
| **433** | 14.9 | 10.8 | 72.8 |
| **439** | 8.8 | 13.6 | 153.7 |
| **539** | 25.2 | 15.5 | 61.4 |
| **540** | 18.2 | 9.4 | 51.3 |
| **549** | 22.4 | 11.4 | 51.0 |
| **627** | 16.2 | 8.7 | 53.7 |
| **638** | 17.0 | 15.3 | 90.0 |
| **642** | 14.8 | 11.4 | 77.1 |
| **662** | 12.4 | 8.6 | 69.4 |
| **665** | 14.2 | 12.0 | 84.4 |
