# Supplementary material for: Isolating Influenza RNA from Clinical Samples Using Microfluidic Oil-Water Interfaces
Source: PLoS One. 2016 Feb 17;11(2):e0149522. doi: 10.1371/journal.pone.0149522 (PMC4757531; doi:10.1371/journal.pone.0149522)
Supplement: S2 Table — (DOC) [file pone.0149522.s005.doc]

Table S2

| Sample ID | Kit C**_t_** | Chip C**_t_** | Kit VL (#/mL) | Chip VL (#/mL) | ΔC**_t_** | E**_vir_** (%) |
| --- | --- | --- | --- | --- | --- | --- |
| **78** | 29.67 | 29.05 | 5.85E+06 | 8.97E+06 | -0.62 | 153.33 |
| **86** | 26.50 | 28.26 | 5.20E+07 | 1.55E+07 | 0.5 | 70.71 |
| **98** | 26.01 | 27.43 | 7.30E+07 | 2.74E+07 | 1.42 | 37.37 |
| **103** | 25.76 | 27.30 | 8.67E+07 | 3.00E+07 | 1.54 | 34.39 |
| **134** | 31.32 | 33.17 | 1.87E+06 | 5.23E+05 | 1.85 | 27.74 |
| **177** | 26.25 | 26.47 | 6.18E+07 | 5.31E+07 | 0.22 | 85.92 |
| **179** | 26.62 | 26.20 | 4.79E+07 | 6.40E+07 | -0.42 | 133.79 |
| **193** | 30.30 | 30.86 | 3.79E+06 | 2.57E+06 | 0.56 | 67.81 |
| **204** | 32.73 | 34.34 | 7.09E+05 | 2.34E+05 | -0.76 | 169.35 |
| **218** | 28.12 | 29.22 | 1.70E+07 | 7.98E+06 | 1.1 | 46.65 |
| **232** | 29.72 | 32.14 | 5.65E+06 | 1.06E+06 | 2.42 | 18.69 |
| **254** | 26.93 | 30.55 | 3.87E+07 | 3.19E+06 | 2.07 | 23.82 |
| **286** | 28.39 | 28.85 | 1.41E+07 | 1.03E+07 | 0.46 | 72.7 |
| **318** | 22.33 | 23.21 | 9.23E+08 | 5.03E+08 | 0.88 | 54.34 |
| **327** | 25.18 | 26.04 | 1.29E+08 | 7.15E+07 | -0.54 | 145.4 |
| **349** | 25.83 | 27.88 | 8.26E+07 | 2.01E+07 | 1.42 | 37.37 |
| **401** | 27.14 | 27.31 | 3.35E+07 | 2.98E+07 | 0.17 | 88.88 |
| **412** | 27.71 | 28.37 | 2.26E+07 | 1.43E+07 | 0.66 | 63.29 |
| **433** | 27.70 | 29.96 | 2.28E+07 | 4.79E+06 | 2.26 | 20.88 |
| **439** | 27.98 | 29.61 | 1.88E+07 | 6.09E+06 | -0.75 | 168.18 |
| **539** | 28.31 | 29.63 | 1.49E+07 | 6.01E+06 | 1.32 | 40.05 |
| **540** | 30.43 | 31.70 | 3.46E+06 | 1.44E+06 | 1.27 | 41.47 |
| **549** | 28.58 | 31.05 | 1.24E+07 | 2.26E+06 | 2.47 | 18.05 |
| **627** | 26.35 | 28.28 | 5.77E+07 | 1.53E+07 | 1.93 | 26.24 |
| **638** | 19.44 | 20.49 | 6.77E+09 | 3.28E+09 | 1.05 | 48.3 |
| **642** | 26.71 | 27.08 | 4.50E+07 | 3.49E+07 | 0.37 | 77.38 |
| **662** | 27.86 | 29.84 | 2.04E+07 | 5.20E+06 | 1.57 | 33.68 |
| **665** | 22.10 | 25.25 | 1.08E+09 | 1.23E+08 | 3.15 | 11.27 |
